# Complete mitochondrial genome of *Palaemonetes sinensis* and its phylogenetic consideration

**DOI:** 10.1080/23802359.2019.1693934

**Published:** 2019-11-22

**Authors:** Rui Li, Xi-Dong Mu, Chuan-Jie Qin, Jun Wang, Yang He, Zheng-Yong Wen

**Affiliations:** a Key Laboratory of Sichuan Province for Fishes Conservation and Utilization in the Upper Reaches of the Yangtze River, Neijiang Normal University, Neijiang, China;; b College of Life Science, Neijiang Normal University, Neijiang, China;; c Ministry of Agriculture and Rural Areas, Pearl River Fisheries Research Institute, Chinese Academy of Fishery Sciences, Key Laboratory of Recreational Fisheries, Guangzhou, China;; d BGI Education Center, University of Chinese Academy of Sciences, Shenzhen, China

**Keywords:** Mitochondrial genome, *Palaemonetes sinensis*, phylogenetic analysis

## Abstract

The aim of this study was to determine the complete mitochondrial genome (mitogenome) and the phylogenetic location of the *Palaemonetes sinensis*. The mitogenome was 15,736 bp in length, containing 22 transfer RNA genes (tRNAs), 13 protein-coding genes (PCGs), 2 ribosome RNA genes (rRNAs), and a control region (CR). The overall nucleotide composition is as follows: A, 35.69%; C, 21.66%; G, 12.39%; T, 30.26%. Nine and four PCGs were encoded on the heavy and light strands, respectively. Phylogenetic analysis suggested that *P. sinensis* shares a close relationship with *Palaemon serenus* and *Palaemon capensis*. These findings are helpful for better understanding the phylogenetic relationship among Caridea, as well as investigating the population genetics of *P. sinensis* in the future.

The Chinese grass shrimp, *Palaemonetes sinensis* (Caridea: Palaemonidae), is a small-scale economic shrimp widely distributed in China and adjacent areas (Li et al. 2018). Because of its bright-colored appearance and appealing flavor, *P. sinensis* becomes an aquarium and a fishery species. However, its natural population has dramatically decreased due to habitat destruction and overfishing. Meanwhile, rare genetic information is available and the phylogenetic status of this species is still limited. Hence, we sequenced its mitogenome and used this to investigate its phylogenetic position.

In the present study, the specimens (accession number: Sh-Ps201701-03) were collected from Panjin city (122°08′24″E, 41°00′24″N) in Liaoning Province, and now stored at the Herbarium of Neijiang Normal University. The mitogenome was sequenced by the next-generation sequencing and assembled according to the reference mitogenome of *P. capensis* (Wood et al. 2017).

The complete mitogenome of *P. sinensis* (GenBank: MN372141) was 15,736 bp in length, which was similar to those of other Palaemonidae species including *P. modestus* (Wang et al. 2017), *P. carinicauda* (Shen et al. 2009), and *P. gravieri* (Kim et al. 2017). The nucleotide composition was 35.69% A, 30.26% T, 21.66% C, 12.39% G, with 54.90% AT, respectively. Meanwhile, 13 protein-coding genes (PCGs), 22 transfer RNA genes (tRNAs), 2 ribosome RNA genes (rRNAs), and 1 control region (CR) were encoded by the circular mitogenome. Only four PCGs (*nad5*, *nad4*, *nad4l*, and *nad1*) were located at the light strand, and all the other PCGs were located at the heavy strand. Additionally, six initiation codons (ATT, ACT, ATG, ATC, ATA, and GTG) and two termination codons (TAG and TAA) were identified. Furthermore, the length variations of tRNAs range from 58 bp (tRNA^Ser^) to 78 bp (tRNA^Glu^). Finally, the gene orders of *P. sinensis* mitogenome were identical to its closely related species.

In order to investigate the phylogenetic position of *P. sinensis*, a phylogenetic tree of Caridea was constructed based on a dataset of the protein sequence of 13 PCGs. As shown in Figure 1, the tree was divided into two groups of Palaemonidae and Alpheidae, and the Palaemonidae group was further clustered into two clades of *Palaemon* and *Macrobrachium*. Meanwhile, the *P. sinensis* was grouped into the *Palaemon* clade and shared a close relationship with *P. capensis* and *P. serenus*.

**Figure 1.**
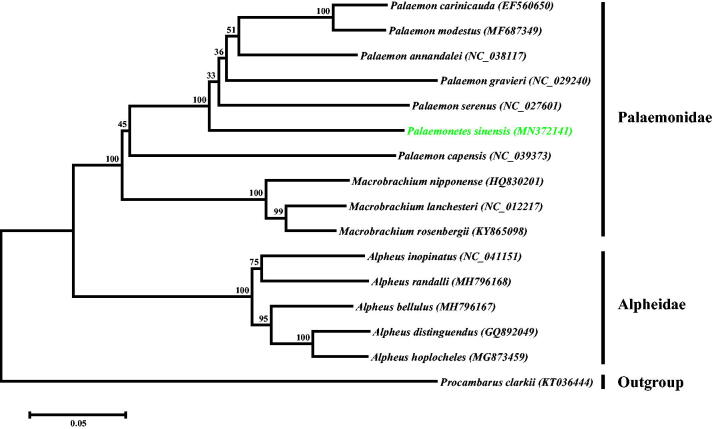
Phylogenetic analysis infers the evolutionary relationship of Caridea. The tree was constructed based on maximum-likelihood method using Mega 6.0 software. *Palaemonetes sinensis* was highlighted by green type, and the *Procambarus clarkii* was used as the outgroup.
